# Speech therapy for transgender women: an updated systematic review and meta-analysis

**DOI:** 10.1186/s13643-023-02267-5

**Published:** 2023-07-23

**Authors:** Karine Schwarz, Carla Aparecida Cielo, Poli Mara Spritzer, Anna Paula Villas-Boas, Angelo Brandelli Costa, Anna Martha Vaitses Fontanari, Bruna Costa Gomes, Dhiordan Cardoso da Silva, Maiko Abel Schneider, Maria Inês Rodrigues Lobato

**Affiliations:** 1 Department of Psychiatry, Gender Identity Program at Hospital de Clínicas de Porto Alegre and Federal University of Rio Grande Do Sul, Porto Alegre, 90035-003 Brazil; 2 Gynecological Endocrinology Unit, Division of Endocrinology, Hospital de Clínicas de Porto Alegre, Porto Alegre, Rio Grande Do Sul Brazil; 3 Department of Speech Therapy, Voice Laboratory, Federal University of Santa Maria, Santa Maria, Rio Grande Do Sul Brazil; 4 Department of Physiology, Federal University of Rio Grande Do Sul, Porto Alegre, Rio Grande Do Sul Brazil; 5 Psychology Graduate Program, Pontifical Catholic University of Rio Grande Do Sul, Porto Alegre, Rio Grande Do Sul Brazil; 6 Clinical Speech Therapist, Osório, Rio Grande Do Sul Brazil; 7 Department of Psychiatry and Behavior Neuroscience, McMaster University, Ontario, Canadá

**Keywords:** Gender dysphoria, Transgender, Gender identity, Clinical care, Voice

## Abstract

**Background:**

We systematically reviewed the literature and performed a meta-analysis on the effects of speech therapy and phonosurgery, for transgender women, in relation to the fundamental frequency gain of the voice, regarding the type of vocal sample collected, and we compared the effectiveness of the treatments. In addition, the study design, year, country, types of techniques used, total therapy time, and vocal assessment protocols were analyzed.

**Methods:**

We searched the PubMed, Lilacs, and SciELO databases for observational studies and clinical trials, published in English, Portuguese, or Spanish, between January 2010 and January 2023. The selection of studies was carried out according to Prisma 2020. The quality of selected studies was assessed using the Newcastle–Ottawa scale.

**Results:**

Of 493 studies, 31 were deemed potentially eligible and retrieved for full-text review and 16 were included in the systematic review and meta-analysis. Six studies performed speech therapy and ten studies phonosurgery. The speech therapy time did not influence the post-treatment gain in voice fundamental frequency (*p* = 0.6254). The type of sample collected significantly influenced the post-treatment voice frequency gain (*p* < 0.01). When the vocal sample was collected through vowel (*p* < 0.01) and reading (*p* < 0.01), the gain was significantly more heterogeneous between the different types of treatment. Phonosurgery is significantly more effective in terms of fundamental frequency gain compared to speech therapy alone, regardless of the type of sample collected (*p* < 0.01). The average gain of fundamental frequency after speech therapy, in the /a/ vowel sample, was 27 Hz, 39.05 Hz in reading, and 25.42 Hz in spontaneous speech. In phonosurgery, there was a gain of 71.68 Hz for the vowel /a/, 41.07 Hz in reading, and 39.09 Hz in spontaneous speech. The study with the highest gain (110 Hz) collected vowels, and the study with the lowest gain (15 Hz), spontaneous speech. The major of the included studies received a score between 4 and 8 on the Newcastle–Ottawa Scale.

**Conclusion:**

The type of vocal sample collected influences the gain result of the fundamental frequency after treatment. Speech therapy and phonosurgery increased the fundamental frequency and improved female voice perception and vocal satisfaction. However, phonosurgery yielded a greater fundamental frequency gain in the different samples collected. The study protocol was registered at Prospero (CRD42017078446).

**Supplementary Information:**

The online version contains supplementary material available at 10.1186/s13643-023-02267-5.

## Background

The voice is an important marker of gender identity; thus, many transgender women (TW) seek the speech therapy clinic for vocal improvement. Studies show that pitch is one of the main markers of gender in the voice of TW and that TW whose voices present a higher fundamental frequency (*f*_o_) are perceived as more feminine [1, 2]. Emerging evidence indicates that to be perceived as female, the voice *f*_o_needs to be at least between 145 and 165 Hz, called the “ambiguous pitch range.” Other aspects of human communication, both verbal and non-verbal, are important in the process of transition to a new gender identity [3].

Changes in vocal resonance can contribute to the perception of a female voice in TW because, in cis women, the frequencies of formants (*F*_m_) are on average 20% higher than in men [4]. This fact is due to the result of anatomical differences (smaller resonance cavities in women) as well as behavioral differences in phonation (more retracted lips, in the form of a smile, and a more anterior tongue position). The importance of vocal resonance characteristics for gender identification is still not entirely clear.

Hormone therapy rarely results in the development of female vocal characteristics due to the irreversible changes in the laryngeal structure that occur during puberty. Thus, the treatment options for vocal feminization in TW are surgery and speech therapy—voice feminization therapy (VFT), alone or combined [3]. However, there is currently no standardized treatment protocol for vocal feminization.

Among several surgical procedures available for altering *f*_o_, those most cited in the literature are open techniques such as the cricothyroid approach and the modified laryngoplasty technique known as feminization laryngoplasty [5], which alter the laryngeal structure, and endoscopic approaches that focus on the vocal folds, such as Wendler glottoplasty and CO_2_laser techniques [3, 6]. These techniques are advised because the production of a female voice with a biologically male vocal organ carries a potential risk for vocal fatigue or trauma to the vocal folds, which would result in a perceptibly tense voice. Open surgical methods are based on three fundamentals: increasing tension, consistency, and decreasing the mass of the vocal folds [7]. However, surgery is not always sufficient to generate a female voice, and these procedures are not without complications [8]. One study found that phonosurgery carried significant risks of complications, such as reduced mean phonation time (61%), pitch instability (1.9%), decreased loudness (1.7 to 6%), vocal fatigue (6%), hoarseness (3%), and dysphonia (1.7%) [9].

Vocal complaints are common in the transgender, nonbinary, and gender-nonconforming (TNG) population [10]. For many TW, VFT is essential for the voice to be perceived as feminine. The focus of speech therapy is usually increasing *f*_o_; however, the satisfaction of individuals with their voices is not necessarily related to pitch. Other characteristics, such as intonation pattern, articulation, resonance, loudness, *F*_m_patterns, tongue placement in the oral cavity, airflow, pragmatics, and the way of speaking, are mentioned as important gender markers and are prescribed in VFT [3, 4].

When VFT is performed without the assistance of a specialized practitioner, the attempt to raise the pitch in a vocal apparatus with male anatomy can cause mild to moderate dysphonia or vocal tension. In addition, other aspects, such as vocal health, breathing exercises, and relaxation must be addressed, because TW is exposed to the same vocal risk behaviors of cisgender people, such as incorrect vocal use [3].

The benefits of VFT before and after surgery combined with the Wendler glottoplasty technique were reported in a study with 10 TW. All experienced significantly increased *f*_o_(mean increase 106 Hz), as well as significant improvements in the degree of voice feminization and self-reported satisfaction [11]. However, few studies describe the types of techniques used, their frequency and timing, the effects of these techniques on the *f*_o_, and voice quality outcomes, as well as which vocal techniques are most effective alone or in conjunction with vocal surgery.

Researchers report that VFT and behavioral changes are of great relevance in the TW voice transition process and that surgery can be indicated as an additional [3]. In addition, they suggest the need for rigorous studies to investigate the most effective methods for the vocal treatment of this population.

In a meta-analysis [9], concluded that both VFT and phonosurgery are efficient, depending on the individual need to increase *f*_o_, cost, and complications of the procedures. The authors did not analyze the types of vocal samples collected. Another study [12] report that the evidence for the effectiveness of VFT is still limited and that there is a lack of rigorous research to determine best-practice guidelines.

Authors [13] cited seven studies, before 2020, that provide empirical evidence of the effectiveness of voice training for TW, although still weak. Overall, voice training methods were similar but effective in increasing mean *f*_o_, *f*_o_ range, vocal satisfaction, self-perception and listener perception of vocal femininity, voice-related quality of life, and social and professional participation. However, there is a lack of randomized controlled trials, small sample sizes, inadequate long-term follow-up, lack of control groups, and control of confounding variables. In addition, the last edition of the Standards of Care [13] recommends that health professionals who intend to work with transgender and gender-diverse people receive education to develop skills in supporting vocal functioning, communication, and well-being of this population; develop appropriate intervention plans for individuals dissatisfied with their voice and communication; and provide pre-and/or post-operative support.

Current studies [14, 15] provide more robust evidence of the effectiveness of speech therapy, despite the feminization of the voice for TW remains a challenge for professionals due to the lack of standardization of protocols, evaluation measurement, and effectiveness of the vocal techniques used. In a retrospective study [14] of 16 cases on the effects of a voice and communication modification program for TW, the results indicated that individuals showed a significant improvement in subjective results, even with small changes in acoustic measurements and vice versa. Another study [15] provided evidence that gender-affirming voice training for TW clients can be effective, both in the intensive and traditional form, in relation to acoustic measurements and vocal satisfaction; however, the training was not sufficient for all participants to reach their goal to develop a consistent feminine voice. Results from other research [16] agree with previous studies with continued targeting of *f*_o_ and vocal tract resonance in voice and communication feminization/masculinization training programs, and provide preliminary evidence for more emphasis on vocal intensity and speech rate and for the importance of non-verbal communication targets in voice training programs and gender-affirming communication [17]. In addition, there are reports of the increase and effectiveness of online and/or hybrid care [18, 19].

Within this context, the underlying research questions of this systematic review were as follows: “What are the methods used in VFT for TW? What are the effects of VFT on the voice? What is the most effective approach (VFT or phonosurgery) concerning *f*_o_ gain and type of sample voice?” Does the VFT time influence the *f*_o_ gain?

## Materials and methods

### The ethical approval statement

This paper is part of the project “A Voz na Disforia de Gênero”, approved by the ethics committee of Hospital de Clínicas de Porto Alegre, Brazil, under number: 04075/2014. The study protocol was registered at PROSPERO (CRD42017078446).

### Search strategy and study selection

In January 2023, two independent raters (KS and APVB) carried out a search of the PubMed, Lilacs, and SciELO databases for articles on the topic. The search strategy consisted of a combination of Medical Subject Headings (MeSH) descriptors and relevant keywords. The keywords used for the search were as follows: ((Transgender Persons) or (Health Services for Transgender Persons) or (Transsexualism) or (Gender Identity)) and ((Voice Training) or (Voice) or (Voice Quality)). The searches were adjusted to meet the requirements of each electronic database. The following filters used: studies published in the last 13 years (2010.01.01 to 2023.01.01).

**Fig. 1:**
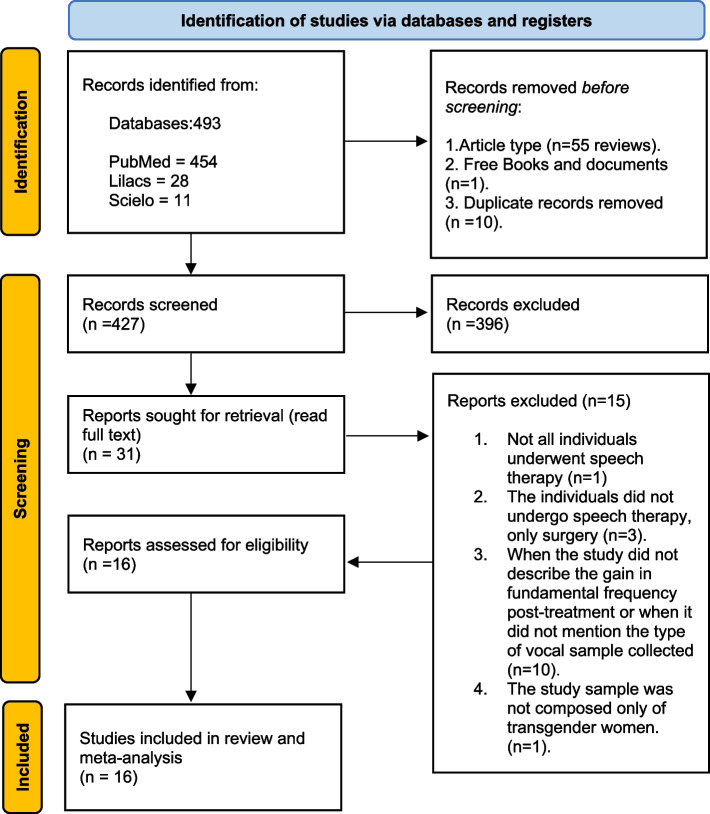
Flow diagram of study selection

According to PRISMA guidelines (2020) (Fig. 1), the selection of articles followed pre-established inclusion and exclusion criteria and a defined PICO question. Articles with the following characteristics were included as follows: design, observational studies (case–control and cohort) and clinical trials; participants (P), TW; intervention (I), VFT; control group (C), phonosurgery (VFTC) or no intervention; and outcome (O), *f*_o_ and type of the sample voice.

### Data extraction and quality control assessment

The inclusion criterion was articles in Portuguese, English, or Spanish, from the last 13 years, and, according to the PICO questions, having the theme VFT or VFTC, in TW, which contained the result of the *f*_o_ gain after the treatments and described the *f*_o_ collection method.

The study exclusion criteria were as follows: (1) when individuals in the treatment groups did not undergo speech therapy, only surgery; (2) when not all individuals in the study group or control group underwent VFT; (3) when the study did not describe the gain of the *f*_o_ of the voice post-treatment or when it did not mention the type of vocal sample collected; and (4) when the study sample was not composed only of TW.

The selection of studies consisted of four stages, with the following hierarchy of eligibility: (1) We removed the reviews and books, (2) duplicates were removed, (3) screening of articles according to the PICO question by reading the title and abstract, and (4) full-text reading by two study researchers (KS) and (APVB) and application of exclusion criteria. The flowchart in Fig. 1, according to PRISMA (2020) guidelines, shows the selection of study articles.

A descriptive analysis of the selected articles began with the application of a protocol prepared by the researchers, designed to collect the following data: authors, year of publication, country, study design, sample size and age, type of procedure, VFT methods, time-course variables of therapy, evaluations performed, results, means of measuring *f*_o_, pre- and post-*f*_o_ data, and final *f*_o_ gain, according to the type of the sample collected.

The methodological quality of the studies was assessed by three previously trained, independent reviewers using the Newcastle–Ottawa Scale (NOS) (KS, APVB, BG). The methodological quality score of the cohort and case–control studies was calculated in three components: selection of the groups (0–4 points), quality of adjustment for confounders (0–2 points), and evaluation of exposure after the outcome (0–3 points). The maximum score is 9 points, which represents high methodological quality.

### Statistical analysis

The mean difference of *f*_o_ gain, with a 95% confidence interval (CI), was estimated using a random effects model. We assessed heterogeneity between studies with *I*^2^ > 50% suggesting moderate heterogeneity and *p* < 0.10 on Cochran’s *Q* test indicating significant heterogeneity [20]. The following variables of interest were included in the meta-analysis: to compare the *f*_o_ gain, in relation to the type of vocal sample collected (vowel, reading, or spontaneous speech), as well as the effectiveness between treatments, subgroup analysis was used. To evaluate the influence of treatment time (in sessions) concerning voice *f*_o_ gain, meta-regression analysis was used. A *p* value of less than 0.05 was considered statistically significant. Statistical analyses were performed using R version 4.2.0 (http://www.r-project.org). The meta package (version 5.2–0) for doing meta-analysis was used within the R environment.

## Results

### Qualitative data synthesis

#### Characterization of the studies

Table 1 shows the year of publication of the studies included in the review and meta-analysis varied between 2012 and 2022, with a predominance of the year 2021, there is an exponential growth graph in recent years. Regarding the country of origin of the studies, the United States of America (USA) was the author who published the most on the subject.

**Table 1:** Description of the results about the treatments (VFT and phonosurgery)

Autor (year) and country	Treatment	Therapy time—session (SD) (min–max)	Age average (years) (SD) (min–max)	Therapy or surgery method	Assessment protocol	Acoustic analysis program	Main results
1. Hancock & Garabedian (2013) [21] USA	Voice feminization therapy	22 SD 18 (2–77)	43 (3.7) (21–60)	Phonotraumatic behaviors, vocal hygiene, relaxation techniques, fundamental frequency, intonation, resonance, vocabulary, pragmatics, nonverbal communication, and respiration	Transgender Self-Evaluation Questionnaire (TSEQ), Perceptive Auditive (CAPE-V and GRBAS)	Visi-Pitch PENTAX Medical	Increased *f*_o_ and improvement of resonance
2. Gelfer & Van Dong (2013) [22] USA	Voice feminization therapy	12 (two 1-h sessions per week for 6 weeks (12 h)	SG: 43.1 (32:11–50:5) CG: women 42. 4 (38:1–44:11) men: 43.11 (38:9–46:8)	Symptomatic voice treatment plus Stemple’s vocal function exercises	Perceptual analysis (CAPE-V)	Real-Time Pitch and multi-speech PENTAX Medical	Better perception of the listener’s vocal femininity
3. Gelfer & Tice (2012) [23] USA	Voice Feminisation Therapy	15 (13–16)	SG: 46.5 42:5 to 52:3 CG: male 46.4 38:8 to 53:8 CG: female 46 40:0 to 51:5	Gelfer method (focus on pitch, quality, intonation, and pitch range)	Semispontaneous Q/A sets) and masculinity and femininity rating scale	Multi-dimensional voice program, real-time Pitch module of the multi-speech PENTAX Medical	Gender identification improved by 1.9%, vocal femininity improved 50.8%, and 33.1% of the time in the long-term
4. Chadwick et al. (2022) [14] USA	Voice Feminisation Therapy	12 weeks (1 session per day)	31.5 (15.5) 18 to 70	Education on vocal hygiene, laryngeal relaxation, implement vocal warm-up exercises, gender-affirming pitch exercises and intonation strategies, resonant voice techniques, gender-affirming volume techniques, articulation techniques, gender-affirming word choices and generalize all gender-affirming techniques	TWVQ	Praat	Significant improvement in TWVQ; significant increases of *f*_o_ in both spontaneous speech and reading, and a significant increase in the frequency range of spontaneous speech. Changes in the acoustic measures did not correlate significantly with changes in TWVQ
5. Quinn et al. (2022) [15] Australia	Voice feminization therapy	Traditional group (one 45-min session per week over 12 weeks), (intensive group) three 45-min sessions per week over 4 weeks	Intensive Group (22–47, Media = 31) Traditional group 21–52, media = 33	Elevate speech pitch and feminize vocal resonance, maintaining vocal health and maximizing vocal strength, flexibility and efficiency. Education and advice on vocal health and voice production; exercises to improve respiratory support and diaphragmatic breathing; head, neck and torso stretches; vocal warm-up/semi-occluded vocal tract exercises; vocal function exercises; and resonant voice exercises, regular practice at home, and structured transfer tasks outside the clinic to facilitate generalization	Voice Quality Index v.02.06 (AVQI)	Praat	Both training programs were effective, with significant positive change in relation to the auditory-perceptual and acoustic evaluation of the voice, greater vocal satisfaction and congruence between gender identity and expression, and reduction of the negative impact of voice concerns in everyday life
6. Brown et al. (2021) [24] USA	Phonosurgery	Pré-surgery VFT 4.6 sessões (1.6) VFTWG 5.0 (1.8) Post-VFTWG 1.8 (1.6)	VFT 35.6 (14.2) VFTWG 35.5 (9.5)	Therapy sessions targeted oral and nasal resonance, feminine speaking patterns, improved voice efficiency, and body language WG	TWVQ, VHI-10, spontaneous cepstral peak prominence, and cepstral spectral index of dysphonia	Computerized speech lab PENTAX Medical	VFT results in *f*_o_ elevation and improvement in VHI-10. The addition of glottoplasty to VFT results in further improvements in *f*_0_ and VHI-10 and statistically significant improvement in TWVQ
7. Casado, Connor, Ângulo and Adrian (2016) [25] Spain	Phonosurgery	24 (2 a 3 session weekly, over 8 weeks)	39.9 (30–52)	Wendler glotoplasty; vocal therapy: vocal hygiene, breathing, relaxation, modulation, emission, projection and modulation, maintenance, and generalization	TMF, TSEQ, VHI, and auditory perceptual analysis (visual analog scale)	Praat	Increased of *f*_o_ higher TMF, better vocal quality self-perception, and listener perception as a more feminine voice
8. Kim (2017) [26] South Korea	Phonosurgery	NA	34.4 (17–63)	Vocal fold shortening and retrodisplacement of the anterior commissure and Speech therapy: laryngeal relaxation, cricothyroid dominant production exercises, resonance modification exercises, exercises to form female formants	Subjective and perceptual assessments, aerodynamic, and acoustic assessments	Real-time pitch PENTAX Medical	Voice femininity and *f*_0_ increased
9.Casado, Parra & Adrian (2017) [11] Spain	Phonosurgery	24 (2–3 sessions weekly over 8–12 weeks) for 60 min each session	SG: 40 (1.6), (30–51); CG: 40.6 (1.8) (36–52)	Wendler’s glotoplasty; speech therapy: basic information and advice, relaxation, respiration training, emission, placement, and modulation of the masculine larynx to feminine tones, maintenance and generalization of newly learned methods to real-life situations	TSEQ, MPT, and auditory perceptual assessment (GRBAS); visual analog scale	Praat	Increase vocal tone and feminization of voice
10. Meister et al. (2016) [27] Germany	Phonosurgery	Before surgery 10: 0–50 sessões After 0–150 sessões	43 (24–57)	Modified Wendler glotoplasty and conservative voice therapy	Dysphonia severity index. VHI, Life Satisfaction Questionnaire, and a visual analog scale of vocal satisfaction	DiVAS 2.4.53 of XION (XION-medical, Berlin, Germany)	Elevation of the vocal pitch for all patients except for one, no correlation between the elevation of *f*_0_ and the number of conservative voice therapy sessions
11. Mastronikolis et al. (2013) [28] Greece	Phonosurgery	NA	37.6 SG: 28.6 (16–39) CG: 51.9 (45–59)	Wendler glotoplasty; vocal therapy. All individuals before surgery and all but two resumed speech therapy after	MPT, phonation quotient, and auditory perceptual analysis (GRBAS) and VHI	Multi-dimensional voice program, PENTAX Medical	Increased of *f*_o_ with younger individuals showing more significant improvement of auditory-perceptual assessment
12. Kelly et al. (2019) [29] Sweden	Phonosurgery	NA	45.5 (10) (35–67)	Wendler glotoplasty and cricothyroid approach; vocal therapy (before surgery): pitch modification trying to reach a female pitch directly or by a step-by-step, oral resonant therapy, accent method and semioccluded vocal tract exercises; (after surgery): mainly on easy and relaxed phonation, vocal hygiene education, keeping the speaking pitch in a female range, and varying the pitch to prevent a limited voice range	Vocal satisfaction questionnaire	Soundswell and Phog (Neovius Data och Signalsystem AB, Lidingö, Sweden	Patients were satisfied at follow-up and rated the GP outcomes more favorably than CTA
13. Thomas et al. (2013) [5] USA	Phonosurgery	NA	Voice feminization laryngoplast: 43 (22–64) feminization laryngoplasty including thyrohyoid approximation: 41 (22–69)	Voice feminization laryngoplasty feminization laryngoplasty including thyrohyoid approximation; voice education discussion. The degree, type, and amount of voice therapy were uncontrolled	Acoustic analysis (*f*_o_ and *f*_o_ range, Hz, and semitone)	NA	Increased *f*_o_
14. Anderson (2014) [30] Canada	Phonosurgery	NA	42 (25–58)	Anterior web formation with injection augmentation. Vocal therapy pré-surgery, without details	Acoustic analysis (*f*_o_ and *f*_o_ range in Hz and semitone). Perturbation measures (jitter and shimmer)	Multi-dimensional voice program, real-time pitch PENTAX Medical	Increased *f*_o_
15. Aires et al., (2021) [31] Brazil	Phonosurgery	Before 24.6 (range 13–54 sessions) 5.7 months	35.4 (27–45)	Wendler glotoplasty NA	TWVQ, self-perceived femininity of the Voice, analysis of CAPE-V protocol phrases GRBAS scale	Voxmetria and fonoview (CTS Play)	*f*_o_, there was a mean increase of 47.9 ± 46.6 Hz (*p* = 0.023); MPT, jitter, shimmer, frequency range, F1 and F2, there was no statistical significance in the pre- and postoperative comparison
16. Casado-Morente et al (2022) [32] Spain	Phonosurgery	At least 10 speech therapy sessions. At 6 months after surgery	GP 38.25 (12.18) VFSRAC + LAVA 34.70 (9.45)	NA	TWVQ and the perceptual assessment using a visual analog scale (PAVAS)	Praat	Both surgical techniques produce the shortening of the vocal folds through an endoscopic approach and result in voices with higher vocal pitch. VFSRAC technique followed by postoperative speech therapy could be recommended for trans-women who wish to feminize their voice

Legends: *f*_*o*_ fundamental frequency of voice. *GP* glossoplasty, *VFTWG* voice feminization therapy plus Wendler glotoplasty, *VFSRAC* + *LAVA* vocal fold shortening, and retrodisplacement of the anterior commissure (VFSRAC) associated with laser assisted voice adjustment (LAVA) cordotomy, *TWVQ* Trans Woman Voice Questionnaire, *VHI* Voice Handicap Index, *Perceptive Auditive Voice protocols* (CAPE-V, GRBAS), *MPT* maximum phonation time, *NA* not available, *SD* standard deviation, *SG* study group, *CG* control group

Of the 16 studies included (Table 1) in the systematic review, six studies performed VFT [14, 15, 21–24] in the study group; among these, two carried out case–control studies, but the control group was without intervention. Others carried out cohort studies, retrospective and prospective, with and without a control group. The authors [15] compared two intervention groups with VFT (traditional and intensive) and investigated [24] the difference between VFT and phonosurgery (see Additional file 1).

As for phonosurgery, five studies were retrospective [25, 26, 30, 32] or prospective [31] cohorts and the other five were case-control [5, 11, 27–29] studies with a comparison of treatment results with different surgical techniques, with or without speech therapy, to groups of different ages (see Additional file 2).

#### Total therapy time

The total therapy time (Table 1) consisted of 5 to 84 sessions, one to three times a week, with an average duration of 1 h. Five studies [5, 26, 28–30] did not report the duration of therapy. In a study, patients received an average of 15 one-hour VFT sessions, twice a week; vocal changes persisted partially for 15 months after the end of therapy [23]. Another performed Wendler glottoplasty plus 24 post-surgery VFT sessions and reported a *f*_o_gain of 106 Hz [25]. One study used intensive therapy (three 45-min sessions per week over 4 weeks) [15].

#### Speech therapy

Regarding VFT exercises, studies have recommended guidelines on phonotraumatic behaviors and vocal hygiene, relaxation techniques, and respiration training [21, 25]; Stemple Vocal Function Exercises [22, 23], with a focus on pitch, quality, intonation, and pitch range; laryngeal relaxation, cricothyroid dominant production exercises, resonance modification exercises, and exercises to form female *F*_m_ [26]. Some studies performed speech therapy only before surgery [30], and some did not mention the details of the techniques employed [27, 31, 32]. Information about the duration of speech therapy, as well as the techniques used and programs used for the collection of *f*_o_data, were lacking from many phonosurgery studies. Current studies recommend that speech therapy be focused on oral and nasal resonance, female speech patterns, improved voice efficiency, and body language [24]. In addition, the authors recommend exercises to maximize respiratory support with diaphragmatic breathing; head, neck, and torso stretch; vocal warm-up/semi-occluded vocal tract exercises; vocal function exercises; and resonant voice exercises, regular practice at home, and structured transfer tasks outside the clinic to facilitate generalization [15] and intonation strategies [14] (Table 1).

#### Vocal assessment

As for vocal assessment, some studies performed an acoustic voice analysis through a collection of the *f*_o_ and administration of self-assessment questionnaires, with a predominance of the Transgender Woman Voice Questionnaire (TWVQ) [14, 24, 31, 32] and perceptual auditory analysis of voice using the GRBAS scale. The acoustic analysis programs differed between studies, with the multi-dimensional voice program by Kay Pentax being the most popular (Table 1).

### Meta-analysis results

#### Gain of *f*_o_ post-treatment and the type of vocal sample collected

**Fig. 2:**
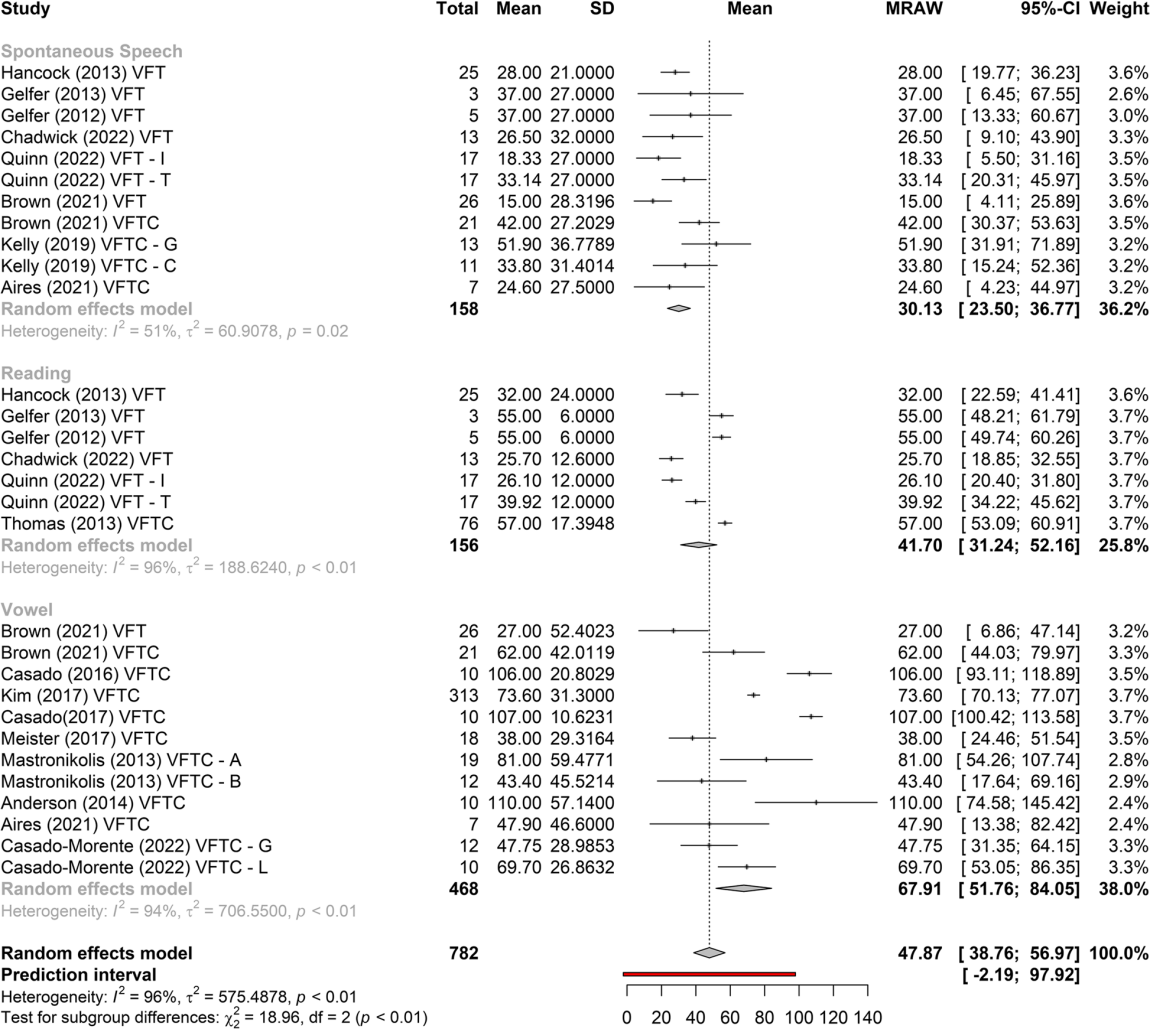
Meta-analysis of the *f*_o_ gain in the VFT, in relation to the types of samples collected. Legends: VFT, voice feminization therapy; VFTC, phonosurgery; VFT–I, voice feminization therapy – intensive; VFT–T, voice feminization therapy – traditional; VFTC–A, phonosurgery with group A; VFTC–B, phonosurgery with group B; VFT–G, voice feminization therapy plus glottoplasty; VFT–C, voice feminization therapy plus cricothyroid approximation; VFT–L, VFSRAC + LAVA—retrodisplacement of the anterior commissure (VFSRAC) associated with laser-assisted voice adjustment (LAVA) cordotomy

There is a difference between the gain of *f*_o_ and the type of vocal sample collected (Fig. 2).

**Fig. 3:**
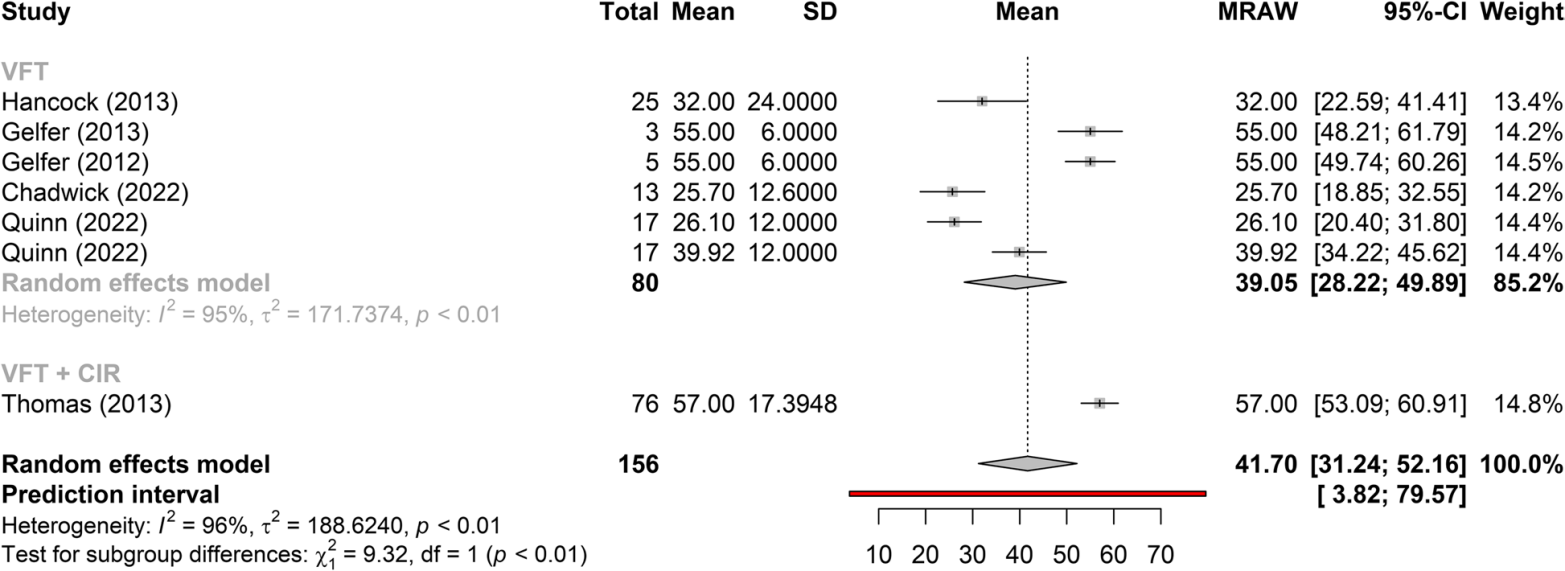
Comparison of the effectiveness of treatments (reading)

**Fig. 4:**
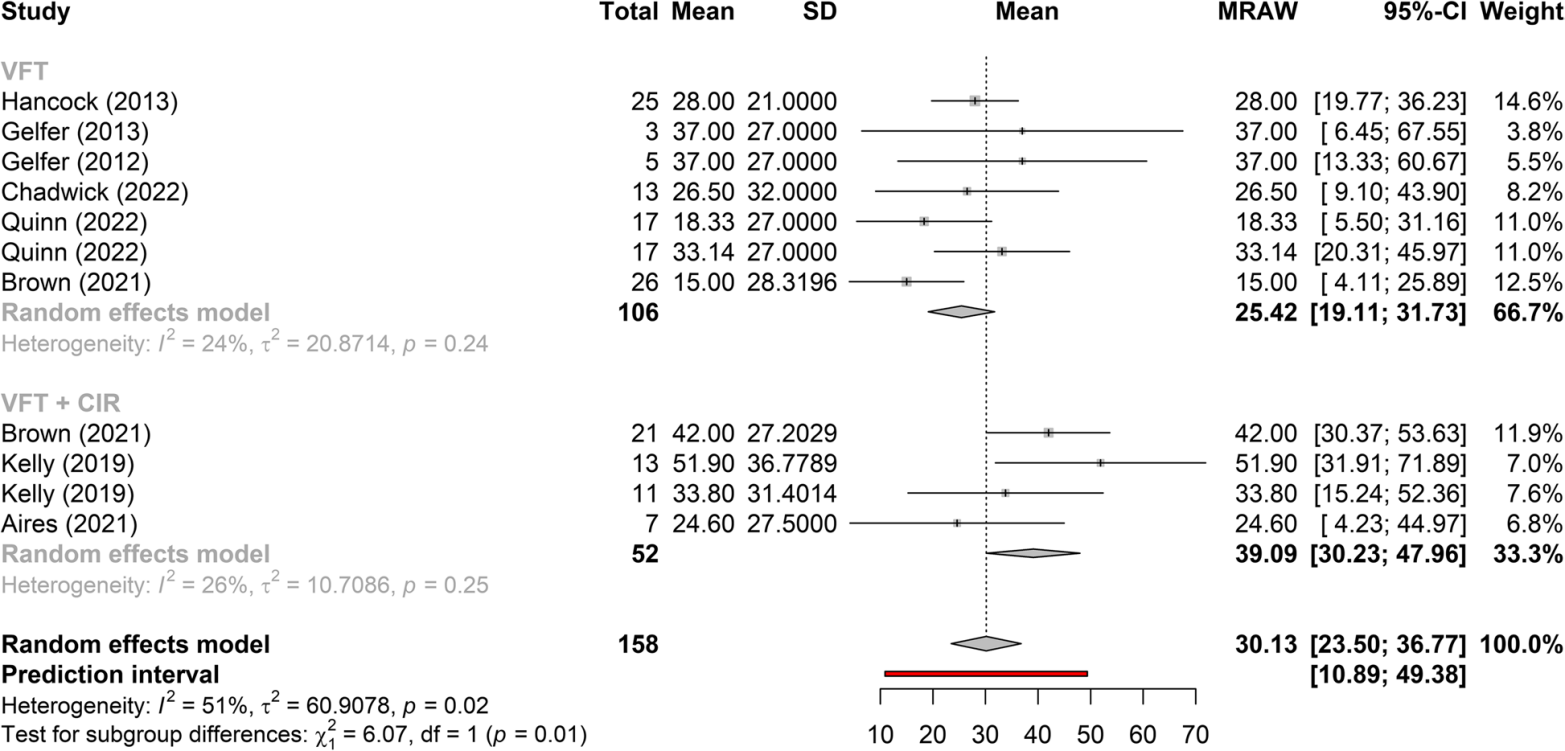
Comparison of the effectiveness of treatments (vowel /a/)

**Fig. 5:**
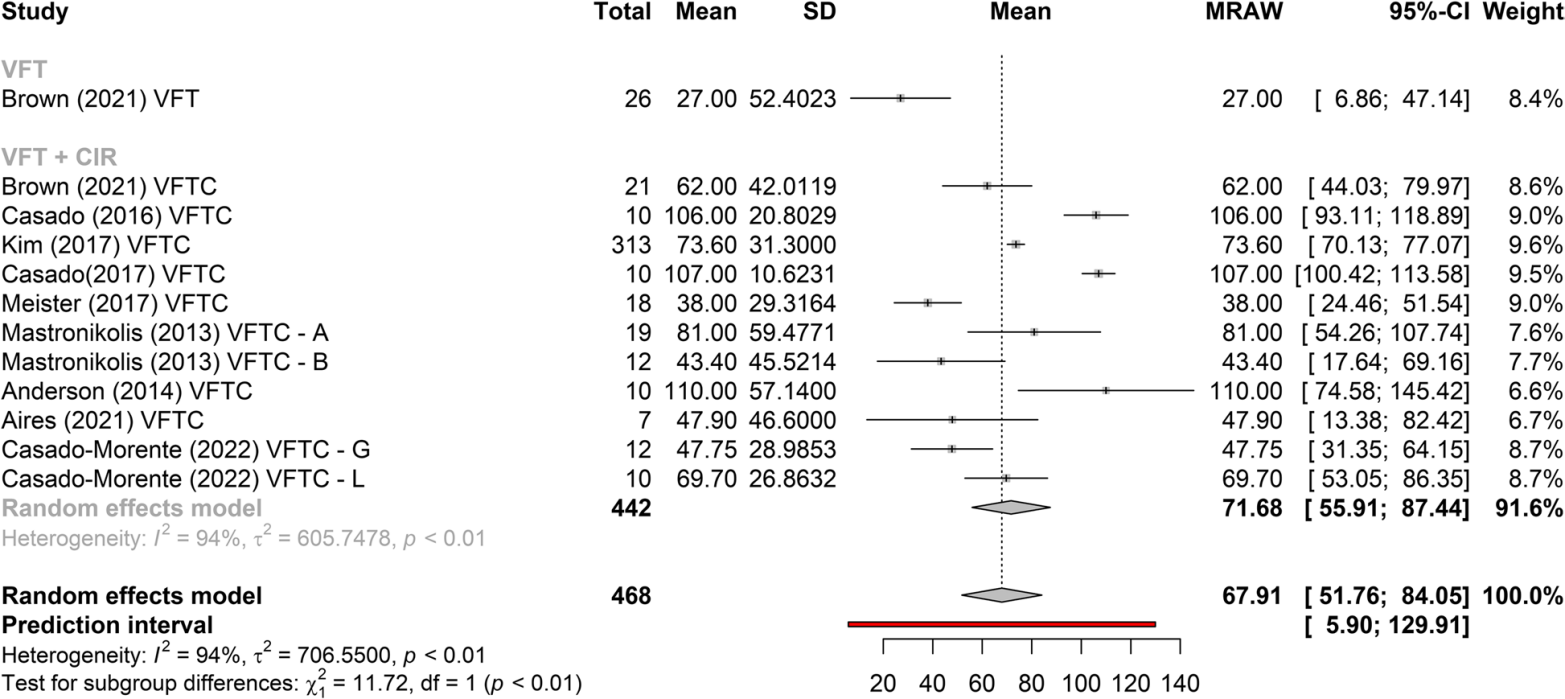
Comparison of the effectiveness of treatments (spontaneous speech)

Figures 3, 4, and 5 show the greatest gain obtained in *f*_o_ post-treatment refers to phonosurgery, that is, phonosurgery is the most effective treatment, independent of the collected sample (*p* < 0.01). As for the gain of *f*_o_, one study about anterior glottic web formation assisted by temporary injection augmentation [30] obtained the highest gain (110 Hz) and another [11] reported greater gain when speech therapy was combined with surgery (107 Hz) than with surgery alone (76 Hz) (see Additional file 2 and Fig. 2).

Among the studies that performed phonosurgery (see Additional file 2), all reported a significant increase in post-treatment *f*_o_ (*p* < 0.05), regardless of the type of vocal sample collected. The study [30] which as noted above obtained the highest gain (110 Hz), collected vowels, and the study with the lowest gain in *f*_o_ 24 Hz in the control group), used spontaneous speech [31] (Fig. 2).

The main effects on the voice were increased *f*_o_ and vocal satisfaction of the patient, improved quality of life, significant improvement of auditory-perceptual assessment, higher TMF, better self-perception of vocal quality, listener perception as a more feminine voice, and improvement of resonance.

**Fig. 6:**
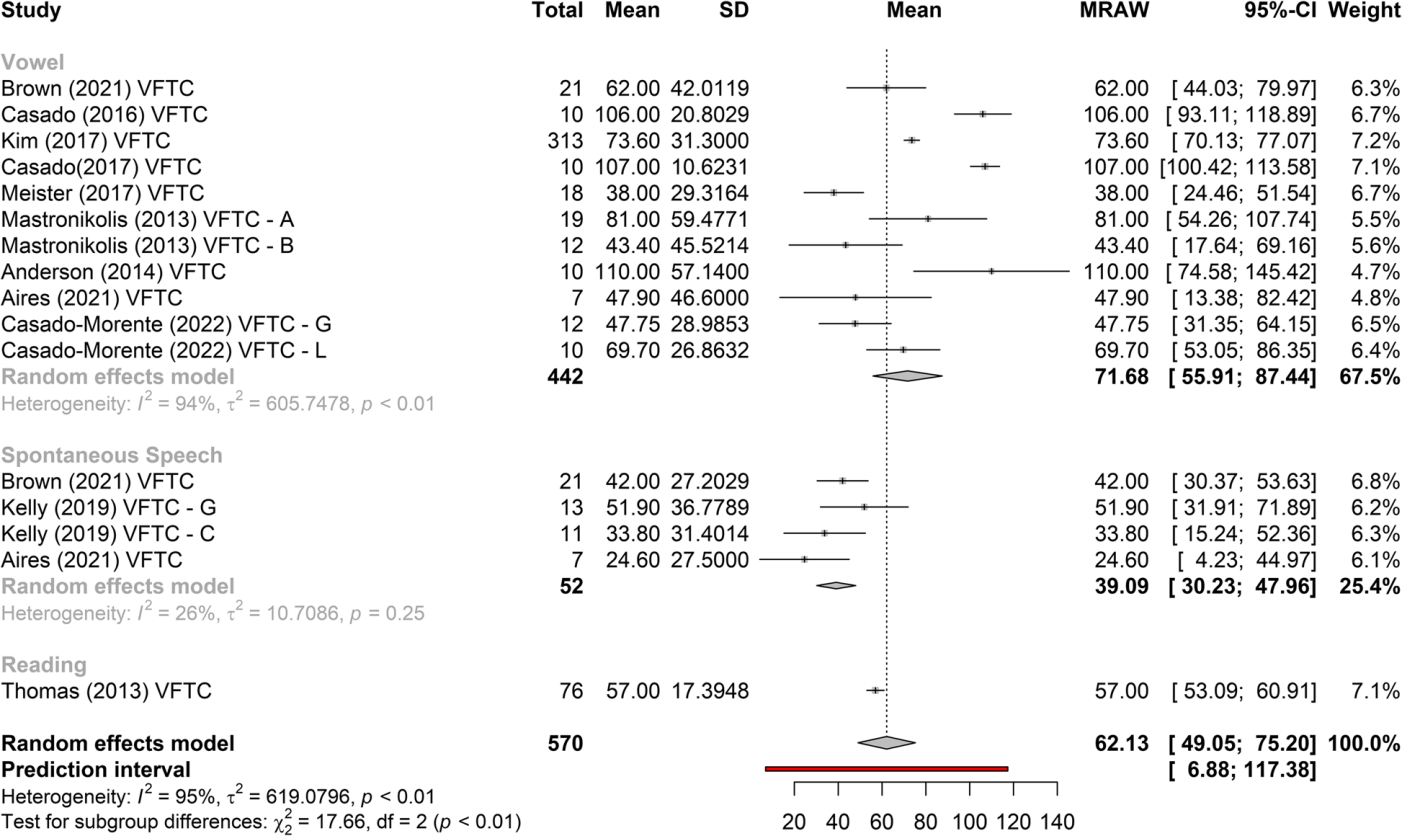
Gain of *f*_o_ in phonosurgery, in relation to the type of vocal sample collected

**Fig. 7:**
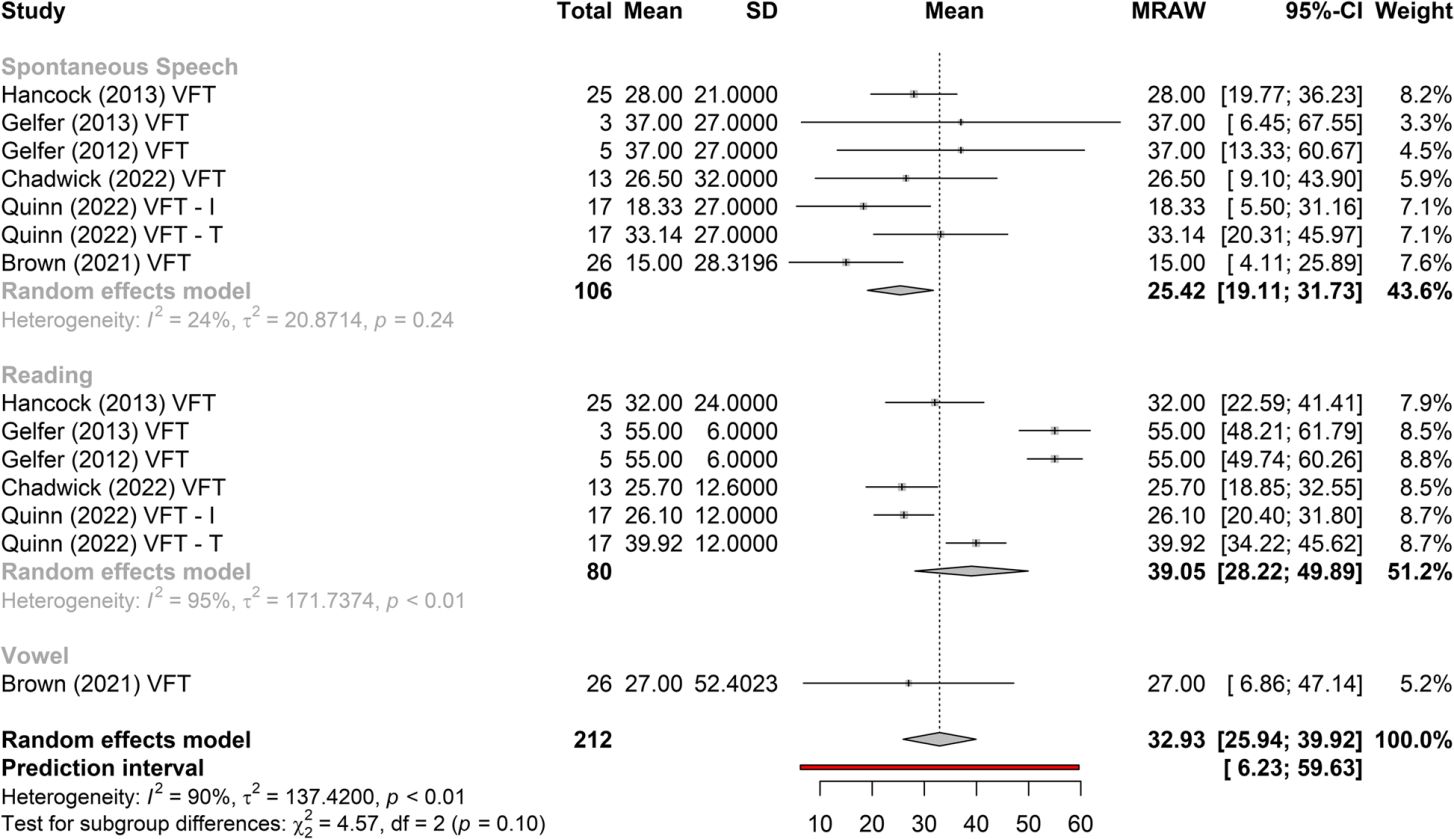
Gain of *f*_o_ in VFT, in relation to the type of vocal sample collected

Two studies [14, 15, 21–24] that evaluated the effects of VFT showed significant gains in *f*_o_, regardless of the type of sample collected (vowel, spontaneous speech, or reading), with less heterogeneity in gain between studies when collected with spontaneous speech (Figs. 2, 6, and 7). The highest gain of *f*_o_with VFT (55 Hz) was found in two studies [22, 23], in the reading sample, and the smallest gain utilizing the spontaneous speech sample (15 Hz) [24] (Fig. 2). A difference of 20 Hz was perceived in another study [21] (higher in vowels and lower in spontaneous speech) and 18 Hz (higher in reading and lower in spontaneous speech, vowel not collected) [23] (Fig. 2).

#### VFT time and the *f*_o_ gain

**Fig. 8:**
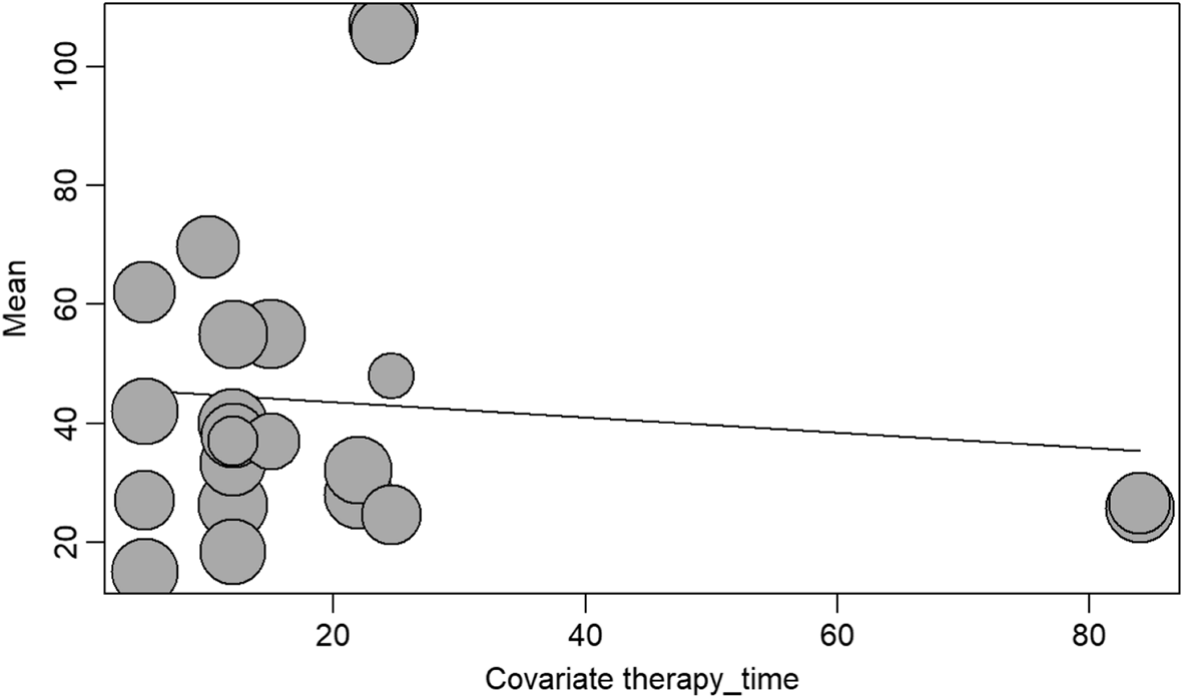
VFT time (in number of sessions) in relation to *f*_o_ gain. Legends: test of moderators (coefficient 2): − 0.1280, (CI 95% − 0.6420, 0.3859); meta-regression *p* = 0.6254

Figure 8 shows that the VFT time did not influence the* f*_o_ gain result. Meta regression *p* = 0.6254, coeficient: − 0.1280 (CI 95% − 0.6420, 0.3859).

### Assessment of methodological quality

Regarding the assessment of methodological quality, Table 2 shows that most of the included studies received a score between 4 and 8 on the NOS. In brief, the studies’ sample sizes were small, there were selection biases regarding control groups, differences in variables of interest (vowel, spontaneous speech, and reading), and some studies did not mention the program used for acoustic analysis or the detailed description of the VFT method. There is a trend toward an increase in studies comparing different treatment methods, with well-defined groups and the same measurements of interest between groups.

**Table 2:** Newcastle-Ottawa Scale (NOS) for quality assessment of studies

Author	Study design	Selection	Comparability	Exposure	Total
1. Hancock and Garabedian (2013) [21]	Cohort	**	*	***	6
2. Gelfer and Van Dong (2013) [22]	Case-control	**	*	*	4
3. Gelfer and Tice (2013) [23]	Case-control	**	*	*	4
4. Quinn et al. (2022) [15]	Cohort prospective	***	**	***	8
5. Brown et al. (2021) [24]	Cohort retrospective	**	**	***	7
6. Chadwick et al. (2022) [14]	Cohort retrospective	**	**	**	6
7. Kim (2017) [26]	Cohort	**	*	***	6
8. Casado, Parra, and Adrian (2017) [11]	Cohort	*	*	***	5
9. Anderson (2014) [30]	Cohort	*	*	***	5
10. Meister et al. (2017) [27]	Case-control	**	**	**	6
11. Casado, Connor, Ângulo, and Adrian (2016) [25]	Case-control	*	*	**	4
12. Kelly et al. (2019) [29]	Case-control	**	*	**	5
13. Thomas and Macmillan (2013) [5]	Case-control	***	*	**	6
14. Mastronikolis et al. (2013) [28]	Case-control	**	*	***	6
15. Aires et al. (2021) [31]	Cohort prospective	***	**	***	8
16. Casado-Morente et al. (2022) [32]	Cohort retrospective	***	**	***	8

*Legends*: Quality of selection (minimum 1; maximum 4 stars); Comparability (minimum 0; maximum 2 stars); Exposure (minimum 1; maximum 3 stars)

## Discussion

This is the first systematic review to analyze the results of VFT and phonosurgery, stratified by different types of vocal samples collected (vowel, reading, or spontaneous speech), and report the evaluation protocols, instruments, and techniques used for the feminization of TW’s voices and the quality of selected studies by Newcastle–Ottawa Scale. Both VFT and phonosurgery resulted in a significant increase in the *f*_o_ of the TW voice; however, phonosurgery provides a significantly greater gain. In both treatment approaches, the smallest gains occurred in the spontaneous speech sample, and even so, the individuals reached the frequency range where the voice could be perceived as female. In addition, the gain results are more homogeneous when spontaneous speech is used. These results provide important guidelines for clinicians who work with the transgender population since vocalizations with reading, automatic sequences, or vowels are situations in which TW can maintain greater self-control of vocal behavior than during spontaneous speech (Figs. 2, 3, 4, 5, 6, and 7).

In spontaneous speech, attention may be more focused on the message and interaction with the listener than on sustaining vowels, automatic speech, or reading; even after phonosurgery, several behavioral adjustments are necessary to ensure vocal adequacy. The focus on the message can divert attention from vocal production and justify the smaller gains in *f*_o_ in a spontaneous speech reported by the different studies analyzed. Thus, it is suggested that isolated or post-surgical speech therapy focuses on prolonging or intensifying learning and the domains of vocal behaviors that facilitate the increase and maintenance of an *f*_o_ closer to that obtained during automatic speech.

While studies of VFT were generally of small sample size and not randomized, the positive results in post-VFT *f*_o_ gains, *f*_o_values at follow-up, and perceptual analyses concerning femininity and masculinity corroborate the indication of VFT. One study notes that the literature supports both VFT and phonosurgery, depending on the magnitude of pitch gain desired by the client, costs, and possible complications. In addition, the authors concluded that VFT provides high vocal satisfaction to customers, is effective at increasing the pitch, and is not invasive [10]. Studies [14, 15, 24] that compared the effects of different methods of speech therapy, for TW, found that the tested methods are effective, with a significant improvement in auditory and acoustic voice perception, greater vocal satisfaction, greater congruence between gender identity and expression and reduction of negative impact on everyday life. Characteristics customers will look for surgery after vocal training are still unknown [13].

In this study, the relationship between therapy time (in sessions) and post-treatment *f*_o_ gain was not significant (Fig. 8). Nonetheless, the authors [22] stated that both the largest number of sessions and the experience of living full-time as a woman can be important variables in predicting the progress of therapy. Another study [33] found that the largest change in *f*_o_ was directly correlated with the increase in the number of vocal training sessions. One study with an average of 22 VFT sessions obtained an *f*_o_ gain of 48 Hz and concluded that the only clear predictor for a result with a higher *f*_o_is the increase in the number of treatment sessions [15]. Another study that reported the highest gain in *f*_o_ (110 Hz), VFT was performed only before surgery, and the duration of therapy was not mentioned (Table 1) [30]. There is a need for further studies on the topic.

Regarding vocal assessment, the studies were uniform in terms of auditory-perceptual assessment through the application of the CAPE-V and GRBAS protocols (Table 1). In this sense, a previous study has shown that the two scales are reliable and indicated for analysis of voice quality; however, GRBAS was classified as the fastest and CAPE-V the most sensitive, mainly to detect small changes in voice, as in the case of TW [34].

Of note, in most of the included studies, acoustic analysis of the voice was performed by extraction of *f*_o_ (in all studies) and measurement of the mean frequency range, perturbation, and noise (mean percentage jitter, shimmer, and noise-to-harmonics ratio). However, differences in study results and difficulties in comparing the analysis of VFT effects concerning those of phonosurgery can also be related to the various programs used in data extraction, forms of recording, environmental noise, or cultural factors that can affect amplitude and *f*_o_ [35]. In addition, the gain was more homogeneous between studies when investigated in the spontaneous speech sample. There was a significant difference between the gain of the studies and the type of collection. In this way, it is suggested a greater standardization in the collection of pre and post-treatment *f*_o_, to compare results and the use of spontaneous speech in the sample collection, as it better translates the patient’s speech and contributes to the generalization of the highest *f*_o_ gain to spontaneous speech (Fig. 2).

In a study that set out to identify which are the predictive parameters of masculinity-femininity ratings by presenting auditory and visual cues for transgender and cisgender, the authors found statistical significance to *f*_o_, average vowel *F*_m_, and sound pressure level [17]. Indeed, a study [36] report that methods for voice feminization are based on changing four parameters: *f*_o_, resonance frequency relative to vocal tract volume and length, *F*_m_ tuning, and phonatory pattern. In the present systematic review, only two studies that analyzed the first three *F*_m_ of /i/ from the word “beach”, the first three *F*_m_ for /i/, /a/, and /u/ (from the isolated vowels); and /i/ from the selected semi-spontaneous Q/A set included *F*_m_analysis [22, 23]. One study showed that the frequencies of forming vowels are important clues to the perception of gender, mainly in the range between 145 and 165 Hz, and more prominent in spontaneous speech than in isolated vowels or syllables [37]. A research [26] used resonance modification exercises to form female *F*_m_ based on the source/filter theory of voice production. Thus, it is important to include an investigation of *F*_m_ through spectrographic vocal analysis in future protocols for voice evaluation in TW.

The most used self-assessment questionnaire among the included studies was the Trans Woman Voice Questionnaire (TWVQ) followed by the Transgender Self-Evaluation Questionnaire (TSEQ) and Voice Handicap Index (VHI) (Table 1). The TSEQ is the oldest questionnaire developed for this purpose [38, 39] and was modified from the VHI for the transgender population. Although TSEQ is widely used by researchers in North America and abroad, there are few studies on its characteristics, and its psychometric properties have not yet been established. Scientific evidence supports the validity and indication of the TWVQ as an important tool for TW to perceive the functioning of their voices and how it impacts their daily lives [40].

Both studies focusing on VFT [21, 23] and those combining it with phonosurgery [11, 25] recommend guidelines on phonotraumatic behaviors vocal hygiene, relaxation techniques, and respiration training, because transgender people are exposed to the same types of vocal misuse behaviors of cisgender people. Some phonosurgery studies did not mention the details of the VFT exercises and recommendations before or after surgery, which makes it difficult to compare the effects of VFT on the voice in relation to phonosurgery.

The Stemple Vocal Function Exercises (VFE) method was used in two studies [22, 23]. Stemple’s physiological vocal therapy is based on anatomy and physiology and seeks to modify the function of the laryngeal musculature and the respiratory support provided for voice production. The approach involves direct modification of inappropriate physiological activity through exercises, which focus on airflow and strength of the laryngeal muscles to balance the breathing, phonation, and resonance systems. A study [23] recommends that the main objective of VFT is the modification of pitch, quality, intonation, and pitch range and started their sessions with the production of the consonant /m/ plus vowels with the new *f*_o_ target for habituation and facilitation of oral resonance. A target *f*_o_ was chosen for each person based on age, vocal range, and initial *f*_o_ measurements. Afterward, words starting with /n/, /l/, and /r/ were introduced, followed by phrases with the objective of training intonation, pitch, and vocal quality, and, finally, multiple sentences involving descriptions of figures, roleplaying, and open-ended questions. Two studies [22, 23] also recommend the use of the *Real-Time Pitch Software* from *Multi-Speech* for immediate feedback on the appropriate frequency and production of smooth voice quality (Table 1).

Among the six studies that performed VFT, three used intonation as one of the focuses of therapy. The authors showed that intonation was the target of VFT in 68% of cases; during VFT, the client should demonstrate an appropriate vocal variety and increase in rising intonation, regardless of the objective of increasing *f*_o_, and practicing with phrases, sentences, and paragraphs [21]. Research is still inconclusive about the use of intonation [41], but it appears that most authors who researched VFT recommend its use. Among the phonosurgery studies, there was no such recommendation, but many studies did not detail the method used in speech therapy (Table 1).

As for resonance exercises, the three studies of VFT and two of phonosurgery recommended this modality. The authors [23] reported that 96% of clients used resonant voice in words with initial-position bilabial and semivowel phonemes, with resonance exercises involving nasal sounds and auditory feedback [21]. Most studies recommend moving resonance forward into the oral cavity (“lip spreading and forward tongue carriage”) as a focus of therapy [11, 23, 29], initially in isolated vowels, consonant–vowel, and vowel-consonant syllables, followed by monosyllabic words, bisyllabic words, phrases, full sentences, and conversation. The other studies did not specify how vocal resonance was targeted.

The results of one prospective study found that the TW voice was perceived as female for vowels that had increased frequencies in the *F*_m_, in addition to an increase in *f*_o_ [42]. In a study [4] on the effectiveness of five oral resonance therapy sessions with 10 TW, targeting lip spreading and forward tongue carriage, showed that both the values of the first *F*_m_ (*F*_1_, *F*_2_, and *F*_3_) of vowels /a/, /i/, and /℧/ and *f*_o_ increased after VFT, with the change in *F*_3_being statistically significant. In addition, most TW were perceived as more feminine by others and through self-assessments and were more satisfied with their voices [4]. Oral resonance therapy appears promising and is consistent with the fact that *F*_m_differs between genders; however, further research is needed to prove the effectiveness of the method for TW. One study recommended the use of vocabulary, pragmatic [28], and two nonverbal communication for feminization [24]. There is no scientific evidence of gender differences in these parameters [28].

As for surgical techniques combined with VFT, Wendler glottoplasty was the most cited, in seven studies with an *f*_o_ gain of up to 107 Hz [11] (see Additional file 2 and Fig. 1). The study [30] obtained the greatest gain in *f*_o_ (110 Hz) with pre-surgery VFT and anterior web formation with injection augmentation. The lowest gain of *f*_o_ was collected through spontaneous speech, using VFT (15 Hz) [24]. A reserach [11] studying the effect of phonosurgery in relation to surgery alone showed that VFT promotes better gain in *f*_o_ when combined with surgery (107 Hz with VFT vs. 76 Hz without VFT), corroborating the results of this study, which showed that phonosurgery is more effective than speech therapy alone, in relation to the gain in *f*_o_. One research reported that endoscopic shortening was more effective in raising *f*_o_, with an increase of more than 70 Hz, versus a change from 26 to 40 Hz achieved by VFT and other surgical options [9].

The authors [3] state that the variations between these studies are mainly due to the complex nature of the individual, differences in vocal demands, and the lack of standardization of vocal feminization treatments among voice practitioners. In addition, the assessment of methodological quality using the NOS showed great variability between studies in how *f*_o_ was collected, which acoustic analysis software was used; sample sizes are small, and there are biases in the selection of control groups, as well as the lack of detailed information about the speech therapy steps and techniques used. This made it difficult to compare results across the included studies.

The limitations of the present review are the small number of published articles, their small sample sizes, and the differences in the *f*_o_ analysis methods of each study. As we verified differences between the different types of collected voice samples, it is not possible to group all the results without considering these differences.

## Conclusions

Both VFT and phonosurgery showed an increase in the *f*_o_ of the voice, regardless of the type of vocal sample collected, with phonosurgery having presented a significative greater gain of *f*_o_. The type of vocal sample collected significantly influenced the result of *f*_o_ gain after treatment. The speech therapy time did not influence the post-treatment gain in voice *f*_o_. The quality of evidence of the studies was low, given the lack of randomized controlled trials, the small sample sizes, and the different methods of collecting *f*_o_. Thus, this study may provide additional evidence on the role of VFT and phonosurgery on voice feminization and encourage further research in the field.

### Supplementary Information

**Additional file 1. **Effects of voice feminization therapy on fo in transgender women Legends: NA: not available; SD: standard deviation; fo: fundamental frequency of voice. * Statistical result found by the authors of the study cited in the comparison of fo gain between the pre and post treatment or Significant effects for therapy time versus fo significant effects for time versus fo 15.

**Additional file 2. **Effects of phonosurgery on fo in transgender women Legends: SD: standard deviation; fo: fundamental frequency; * Statistical result found by the authors of the study cited in the comparison of f0 gain between the pre and post treatment. * Wendler glottoplasty (WG) and its modification, the vocal fold shortening, and retrodisplacement of the anterior commissure (VFSRAC) associated with laser assisted voice adjustment (LAVA) cordotomy. VFT: Voice Feminization Therapy.

## Data Availability

We have one supplementary material.

## References

[1] Gelfer MP, Mikos VA (2005). The relative contributions of speaking fundamental frequency and formant frequencies to gender identification based on isolated vowels. J Voice.

[2] Dahl KL, Mahler LA (2020). Acoustic features of transfeminine voices and perceptions of voice femininity. J Voice.

[3] Gray ML, Courey MS (2019). Transgender voice and communication. Otolaryngol Clin North Am.

[4] Carew L, Dacakis G, Oates J (2007). The effectiveness of oral resonance therapy on the perception of femininity of voice in male-to-female transsexuals. J Voice.

[5] Thomas JP, MacMillan C (2013). Feminization laryngoplasty: assessment of surgical pitch elevation. Eur Arch Otorhinolaryngol.

[6] Kanagalingam J, Georgalas C, Wood G, Ahluwalia S, Sandhu G, Cheesman AD (2005). Cricothyroid approximation and subluxation in 21 male-to-female transsexuals. Laryngoscope.

[7] Remacle M, Matar N, Morsomme D, Veduyckt I, Lawson G (2011). Gottoplasty for male-to-female transsexualism: voice results. J Voice.

[8] Borsel J, Eynde E, Cuypere G, Bonte K (2008). Feminine after cricothyroid approximation?. J Voice.

[9] Nolan IT, Morrison SD, Arowojolu O, Crowe CS, Massie JP, Adler RK (2019). The role of voice therapy and phonosurgery in transgender vocal feminization. J Craniofac Surg.

[10] Kennedy E, Thibeault SL (2020). Voice-gender incongruence and voice health information-seeking behaviors in the transgender community. Am J Speech Lang Pathol.

[11] Casado J, Rodríguez-Parra M, Adrián JA (2017). Voice feminization in male-to-female transgendered clients after Wendler’s glottoplasty with vs. without voice therapy support. Eur Arch Otorhinolaryngol.

[12] Quinn S, Swain N (2018). Efficacy of intensive voice feminization therapy in a transgender young offender. J Commun Disord.

[13] Coleman E, Radix AE, Bouman WP, Brown GR, Vries ALC, Deutsch MB (2022). Standards of care for the health of transgender and gender diverse people, Version 8. Int J Transgend Health.

[14] Chadwick KA, Coleman R, Andreadis K, Pitti M, Rameau A (2022). Outcomes of gender-affirming voice and communication modification for transgender individuals. Laryngoscope.

[15] Quinn S, Oates J, Dacakis G (2022). The effectiveness of gender affirming voice training for transfeminine clients: a comparison of traditional versus intensive delivery schedules. J Voice.

[16] Hardy TLD, Rieger JM, Wells K, Boliek CA (2020). Acoustic predictors of gender attribution, masculinity-femininity, and vocal naturalness ratings amongst transgender and cisgender speakers. J Voice.

[17] Hardy TLD, Boliek CA, Aalto D, Lewicke J, Wells K, Rieger JM (2020). Contributions of voice and nonverbal communication to perceived masculinity-femininity for cisgender and transgender communicators. J Speech Lang Hear Res.

[18] Little CC, Russell S, Hwang C, Goldberg L, Brown S, Kirke D, Courey M. Applications of telemedicine in speech-language pathology: evaluation of patient satisfaction. Laryngoscope. 2022;18. 10.1002/lary.30303. Online ahead of print. 10.1002/lary.30303 35848893

[19] Merrick G, Figol A, Anderson J, Lin RJ (2022). Outcomes of gender affirming voice training: a comparison of hybrid and individual training modules. J Speech Lang Hear Res.

[20] Higgins JP, Thompson SG, Deeks JJ, Altman DG (2003). Measuring inconsistency in meta-analyses. BMJ.

[21] Hancock AB, Garabedian LM (2013). Transgender voice and communication treatment: a retrospective chart review of 25 cases. Int J Lang Commun Disord.

[22] Gelfer M, Dong BRV (2013). A preliminary study on the use of vocal function exercises to improve voice in male-to-female transgender clients. J Voice.

[23] Gelfer M, Tice R (2013). Perceptual and acoustic outcomes of voice therapy for male-to-female transgender individuals immediately after therapy and 15 months later. J Voice.

[24] Brown SK, Chang J, Hu S, Sivakumar G, Sataluri M, Goldberg L (2021). Glottoplasty improves voice therapy outcomes. Laryngoscope.

[25] Casado JC, Connor CO, Angulo MS, Adrian JA (2016). Wendler’s glotoplasty and logopedic treatment in the feminization of the voice in transsexuals: resultados de la valoración pre- vs. poscirugía. Acta Otorrinolaringol Esp..

[26] Kim HT (2017). A new conceptual approach for voice feminization: 12 years of experience. Laryngoscope.

[27] Meister J, Hagen R, Shehata-Dieler W, Kühn H, Kraus F, Kleinsasser N (2017). Pitch elevation in male-to-female transgender persons-the Würzburg approach. J Voice.

[28] Mastronikolis N, Remacle M, Biagini M, Kiagiadaki D, Lawson G (2013). Wendler glottoplasty: an effective pitch raising surgery in male-to-female transsexuals. J Voice.

[29] Kelly V, Hertegård S, Eriksson J, Nygren U, Södersten M (2019). Effects of gender-confirming pitch-raising surgery in transgender women a long-term follow-up study of acoustic and patient-reported data. J Voice.

[30] Anderson JA (2014). Pitch elevation in trangendered patients: anterior glottic web formation assisted by temporary injection augmentation. J Voice.

[31] Aires MM, de Vasconcelos D, Lucena JA, Gomes AOC, Moraes BT (2021). Effect of Wendler glottoplasty on voice and quality of life of transgender women. Otorhinolaryngol.

[32] Casado-Morente JC, Benjumea-Flores FL, Romero-Gómez B, Angulo-Serrano MS, O Connor-Reina C, Casado-Alba C, Galeas-López AJ, Carricondo F (2022). Comparison between two surgical techniques for increasing vocal pitch by endoscopic shortening of the vocal folds. J Voice.

[33] Adessa M, Weston Z, Ruthberg J, Bryson PC. Gender-affirming voice modification for transgender women: characteristics and outcomes. Transgend Health. 2022. Ahead of Print. 10.1089/trgh.2021.0071. 10.1089/trgh.2021.0071 PMC10387149 37525833

[34] Nemr K, Simões-Zenari M, Cordeiro GF, Tsuji D, Ogawa AI, Ubrig MT (2012). GRBAS and Cape-V scales: high reliability and consensus when applied at different times. J Voice.

[35] Carvalho-Teles V, Rosinha A (2008). Acoustic analysis of formants and sound signal disturbance measures in women without vocal complaints, non-smokers and non-drinkers. Int Arch Otorhinolaryngol.

[36] Kim H (2020). Vocal feminization for transgender women: current strategies and patient perspectives. Int J Gen Med.

[37] Gelfer M, Bennett Q (2013). Speaking fundamental frequency and vowel formant frequencies: effects on perception of gender. J Voice.

[38] Davies S, Goldberg JM (2006). Clinical aspects of transgender speech feminization and masculization. Int J Transgender.

[39] Davies S, Goldberg JM. Trans care gender transition: changing speech. Manual of Vancouver Coastal Health, Transcend Transgender Support & Education Society and Canadian Rainbow Health Coalition. 2021. Retrieved from: http://www.vch.ca/transhealth.

[40] Dacakis G, Oates J, Douglas J (2017). Further evidence of the construct validity of the Transsexual Voice Questionnaire (TVQMtF) using principal components analysis. J Voice.

[41] Hancock A, Colton L, Douglas F (2014). intonation and gender perception: applications for transgender speakers. J Voice.

[42] Gallena SJK, Stickels B, Stickels E (2018). Gender perception after raising vowel fundamental and formant frequencies: considerations for oral resonance research. J Voice.

