# Supplementary material for: Speech therapy for transgender women: an updated systematic review and meta-analysis
Source: Syst Rev. 2023 Jul 23;12:128. doi: 10.1186/s13643-023-02267-5 (PMC10363306; doi:10.1186/s13643-023-02267-5)
Supplement: Supplementary file 1 — Additional file 1. Effects of voice feminization therapy on fo in transgender women Legends: NA: not available; SD: standard deviation; fo: fundamental frequency of voice. * Statistical result found by the authors of the study cited in the comparison of fo gain between the pre and post treatment or Significant effects for therapy time versus fo significant effects for time versus fo 15. [file 13643_2023_2267_MOESM1_ESM.docx]

| **Author (year)** | Country | Study Design | \| Study  Group  (TW) \| \| --- \| \| | Control Group | Before  Treatment  *f*_o_ Hz  (SD) | After  Treatment  *f*_o_ Hz  (SD) | *f*_o_ Gain  Hz  (SD) | Sample Collected | p Value* |
| --- | --- | --- | --- | --- | --- | --- | --- | --- | --- | --- |
| 1. Hancock & Garabedian (2013)^21^ | USA | Cohort Retrospective | 25 | none | 122 (29) | 150 (29) | 28 (21) | **Spontaneous Speech** | p=0.000 |
|  |  |  |  |  | 124 (24) | 156 (31) | 32 (24) | **Reading** | p=0.000 |
| 1. Gelfer & Van Dong (2013)^22^ | USA | Case-control | 3 | 3 female  3 male | 136 (46) | 184 (34) | 48 (33) | **Vowel**  **∕a∕** | p=0.000 |
|  |  |  |  |  | 116 | 153 | 37 | **Spontaneous Speech** | NA |
|  |  |  |  |  | 122 (35) | 177 (41) | 55 (6) | **Reading** | NA |
| 1. Gelfer & Tice (2013)^23^ | USA | Case-control (prospective) | 5 | 5 female  5 male | 119 | 178 | 37 | **Spontaneous Speech** | p=0.05 |
|  |  |  |  |  | 123 (35) | 194 (43) | 55 (6) | **Reading** | p< 0.05 |
| 1. Chadwick et al. (2022)^14^ | USA | Cohort Retrospective | 13 | none | 136.3 (12.6) | 162.8 (30.2) | 26.5 (32.0) | **Spontaneous Speech** | p = 0.0131 |
|  |  |  |  |  | 138.1 (13.7) | 163.8 (27.1) | 25.7 (12.6) | **Reading** | p = 0.003 |
| 1. Quinn et al. (2022)^15^ | Australia | Cohort Prospective | Intensive Therapy  Group  (I)  17 | Traditional Therapy  Group  (T)  17 | T  131.85  I  147.7 | T  171.77  I  173.87 | T  39.92  I  26.1 | **Reading** | T  p< 0.000  I  p< 0.001  T2-T3 (training period) |
|  |  |  |  |  | T  130.99  I  141.84 | T  164.13  I  160.17 | T  33.14  I  18.33 | **Spontaneous Speech** | T  p< 0.000  I  p = 0.007  T2-T3 (training period) |
| 1. Brown et al. (2021)^24^ | USA | Cohort Retrospective | 26  VFT | 21  VT with additional glottoplasty (VTWG) | VFT  148 (39)  VTWG  150 (26) | VFT  175 (35)  VTWG  212 (33) | VT: 27  VTWG: 62 | **Vowel**  **∕a∕** | VFT  p=0.017  VTWG  p<.0001 |
|  |  |  |  |  | VFT  133 (19)  VTWG  124 (16) | VFT  148 (21)  VTWG  166 (22) | VFT  15  VTWG  42 | **Speaking Fundamental Frequency** | VFT  p<.0001  VTWG  p<.0001 |
|  |  |  |  |  |  |  |  |  |  |
