# Supplementary material for: Speech therapy for transgender women: an updated systematic review and meta-analysis
Source: Syst Rev. 2023 Jul 23;12:128. doi: 10.1186/s13643-023-02267-5 (PMC10363306; doi:10.1186/s13643-023-02267-5)
Supplement: Supplementary file 2 — Additional file 2. Effects of phonosurgery on fo in transgender women Legends: SD: standard deviation; fo: fundamental frequency; * Statistical result found by the authors of the study cited in the comparison of f0 gain between the pre and post treatment. * Wendler glottoplasty (WG) and its modification, the vocal fold shortening, and retrodisplacement of the anterior commissure (VFSRAC) associated with laser assisted voice adjustment (LAVA) cordotomy. VFT: Voice Feminization Therapy. [file 13643_2023_2267_MOESM2_ESM.docx]

| Author (year) | Country | Study Design | \| Study  Group  (Transgender Women – TW) \| \| --- \| \| | Control Group | Before  Treatment  *f*_o_ Hz  (SD) | After  Treatment  *f*_o_ Hz  (SD) | *f*_o_ Gain  Hz  (SD) | Sample Collected | p Value* |
| --- | --- | --- | --- | --- | --- | --- | --- | --- | --- | --- |
| 7.Casado et al. (2016)^25^ | Spain | Retrospective cohort | 10 | None | 137 (9.8) | 243 (18.35) | 106 | Vowel  ∕a∕ | p=0,005 |
| 8.Kim (2017)^26^ | South Korea | Retrospective cohort | 313 | None | 134.6 (25,2) | 208.2 (37.3) | 73.6 (31.3) | Vowel  ∕a∕ | p=0.0001 |
| 9.Casado, Parra & Adrian (2017)^11^ | Spain | Case-control | 10  Wendler’s Glottoplasty  (WG) + VFT | 8 WG without  VFT (TW) | 136 (8.9) | Only WG  229 (27.06)  VFT + WG  243 (5.80)  212 (9.55) | Only WG  93  VFT + WG  107  76 | Vowel /a/ | Only WG  p<0.000  VFT + WG  p=0.016 |
| 10.Meister et al. (2017)^30^ | Germany | Case-control | 18 (with VFT) TW | 3 (withou VFT) TW | 132 (16.4) | 170 (24.3) | 38 | Vowel  ∕a∕ | p< 0.001 |
| 11.Mastronikolis et al. (2013)^31^ | Greece, Belgium and Italy | Case-control | 19 (Group A) TW | 12 (group B) TW | A  132.8 (41.3)  B  143.8 (5.4) | SG: 213.8 (42.8)  CG: 187 (45.2) | A: 81  B: 43.40 | Vowel  ∕a∕ | A  p= 0.001  B  p=0.003 |
| 12. Kelly et al. (2019)^32^ | Sweden | Case-control | 13 (Glottoplasty = G) | 11 (cricothyroid approximation= C) | G: 118 (11.3) C: 138 (23.7) | G: 170 (35) C: 171 (20.6) | G: 51.9 C: 33.8 | Spontaneous Speech | p< 0.001 |
| 13. Thomas & MacMillan (2013)^5^ | USA | Case-control | 54 (feminization laryngoplasty with thyrohyoid approximation = FLTA) TW | 22 (Thyroid cartilage modifcation= TCM) TW | FLTA: 139  TCM: 139 | FLTA: 196  TCM: 196 | FLTA: 57  TCM: 57 | Reading | p=0.01 |
| 14.Anderson (2014)^27^ | Canada | Retrospective cohort | 10 | None | 127 (21.67) | 238 (59.78) | 110 (57.14) | Vowel  ∕a∕ | p< 0.001 |
| 15. Aires et al. (2021)^29^ | Brazil | Prospective  cohort | 7 | None | 137.7 (24.1) | 185.6 (43.8) | 47.9 ± 46.6 | Vowel  ∕e∕ | *p* = 0.023 |
|  |  |  |  |  | 145.0 (15.5) | 169.6 (29.9) | +24.6 (27.5) | Spontaneous Speech | *p* = 0.029 |
| 16. Casado-Morente et al. (2022)^28^ | Spain | Retrospective  Cohort | 12  Wendler Glottoplasty (G) | 10 VFSRAC  +LAVA (L) | G  129.08  (13.7)  L  131.90 (13.63) | G  176.83 (20.57)  L  201.60 (21.08) | G  47.75  (31.3-64.1)  L  69.7 (53.0-86.3) | Vowel  ∕a∕ | G  p<0.001  L  p<0.001 |
